# Vitality in Newborn Farm Animals: Adverse Factors, Physiological Responses, Pharmacological Therapies, and Physical Methods to Increase Neonate Vigor

**DOI:** 10.3390/ani13091542

**Published:** 2023-05-04

**Authors:** Cécile Bienboire-Frosini, Ramon Muns, Míriam Marcet-Rius, Angelo Gazzano, Dina Villanueva-García, Julio Martínez-Burnes, Adriana Domínguez-Oliva, Karina Lezama-García, Alejandro Casas-Alvarado, Daniel Mota-Rojas

**Affiliations:** 1 Department of Molecular Biology and Chemical Communication, Research Institute in Semiochemistry and Applied Ethology (IRSEA), 84400 Apt, France; c.frosini@group-irsea.com; 2 Agri-Food and Biosciences Institute, Hillsborough BT 26 6DR, Northern Ireland, UK; rmunsvila@gmail.com (R.M.);; 3 Animal Behaviour and Welfare Department, Research Institute in Semiochemistry and Applied Ethology (IRSEA), 84400 Apt, France; m.marcet@group-irsea.com; 4 Department of Veterinary Sciences, University of Pisa, 56124 Pisa, Italy; angelo.gazzano@unipi.it; 5 Division of Neonatology, Hospital Infantil de México Federico Gómez, Mexico City 06720, Mexico; dinavg21@yahoo.com; 6 Facultad de Medicina Veterinaria y Zootecnia, Universidad Autónoma de Tamaulipas, Victoria City 87000, Mexico; jmburnes@docentes.uat.edu.mx; 7 Neurophysiology, Behavior and Animal Welfare Assessment, DPAA, Universidad Autónoma Metropolitana, Xochimilco Campus, Mexico City 04960, Mexico

**Keywords:** domestic animals, vitality, MAS, shivering, BAT, meconium staining

## Abstract

**Simple Summary:**

Vitality is a characteristic that in newborn animals demonstrates their vigor and their general state of health (heart rate, respiratory rate, skin color, time that they take to stand up) during the first hours of life. It can be measured by numerical scores based on some scales made for babies and then adapted for various animals. Vitality can be affected by several factors. The objective of this review is to analyze pharmacological and physical therapies used to increase vitality in newborn farm animals, as well as understand the factors affecting their vitality, such as hypoxia, depletion of glycogen, birth weight, dystocia, neurodevelopment, hypothermia, and finally, the physiological mechanism to achieve thermostability. It is essential to evaluate vitality in newborns because it can contribute to implementing interventions to reduce newborn mortality.

**Abstract:**

Vitality is the vigor newborn animals exhibit during the first hours of life. It can be assessed by a numerical score, in which variables, such as heart rate, respiratory rate, mucous membranes’ coloration, time the offspring took to stand up, and meconium staining, are monitored. Vitality can be affected by several factors, and therapies are used to increase it. This manuscript aims to review and analyze pharmacological and physical therapies used to increase vitality in newborn farm animals, as well as to understand the factors affecting this vitality, such as hypoxia, depletion of glycogen, birth weight, dystocia, neurodevelopment, hypothermia, and finally, the physiological mechanism to achieve thermostability. It has been concluded that assessing vitality immediately after birth is essential to determine the newborn’s health and identify those that need medical intervention to minimize the deleterious effect of intrapartum asphyxia. Vitality assessment should be conducted by trained personnel and adequate equipment. Evaluating vitality could reduce long-term neonatal morbidity and mortality in domestic animals, even if it is sometimes difficult with the current organization of some farms. This review highlights the importance of increasing the number of stock people during the expected days of parturitions to reduce long-term neonatal morbidity and mortality, and thus, improve the farm’s performance.

## 1. Introduction

“Vitality” describes the liveliness or vigor that neonate animals exhibit during the first hours post-parturition [1]. It is a trait that is influenced by several factors, such as intrauterine growth, the physiological immaturity of the newborn, and hypoxia due to dystocia. Moreover, vitality in early postnatal life is affected by the behaviors, maternal factors (e.g., maternal health and nutritional status, maternal care), and environmental conditions during birth [1,2]. Fetuses could be exposed in utero to various deleterious factors that disrupt the blood and oxygen flow through the umbilical cord, often culminating in fetal asphyxia, hypoxia, and metabolic acidosis. These situations are the leading causes of intrapartum and neonatal mortality [3,4,5,6,7]. Prolonged or intermittent asphyxia in utero or during birth physiologically weakens the fetus or newborn and makes them less adaptable to extrauterine life, decreasing their viability and vitality [8,9].

In 1952, Virginia Apgar developed the Apgar scoring system, which provides a method to document the newborn’s condition at specific intervals after birth in humans. This is a valuable objective indicator to determine the effectiveness of resuscitative efforts in newborns. Epidemiologists quickly adopted the Apgar score as an outcome in perinatal research because it is straightforward, easily understood, and almost universally recorded in birth-related data sources. The score comprises 5 categories (skin color, heart rate, reflex irritability, activity/flexion, and respiratory effort) that are each scored from 0 to 2, resulting in an overall range of 0 to 10, with 10 indicating that the highest score was given for each clinical indicator of neonatal well-being. In modern practice, the Apgar score is calculated 1 and 5 min after birth, and again at 10 min if the 5 min Apgar is low [10]. Adapted from the Apgar score, neonatal vitality in farm animals is usually assessed through a numerical score, with heart rate, respiratory rate, mucous membranes coloration, the time it took for the offspring to stand up, and meconium staining signs as measurements [11], and different options and measurements have been used to assess neonatal vitality. The total time of parturition and expulsion of the newborn is considered the most important data [12].

The Apgar score has been adapted by diverse authors for pigs [13,14] and ruminants [15,16]. Unlike the human Apgar score, bovine tests combine a variety of indicators divided into three groups: (1) Clinical signs observed on physical examination; (2) Behavior of calves at birth; (3) Blood tests (to evaluate glucose and cortisol levels) [15,17,18].

Proper vitality assessment is essential for newborn survival in humans and domestic animals. The evaluation of the physiological status of the offspring requires trained personnel, adequate equipment, and timely intervention to correct cardiorespiratory depression or resuscitate the newborn when necessary [13,14]. In many farm animal species, it is essential to have trained assistants on farms or in veterinary clinics and adequate physical resources to assess the vitality of the newborn. Early recognition of dystocia and fetal distress is essential for successfully managing labor and neonatal health [19].

Currently, several methods and therapies are applied to farm animals to assess and improve the vitality of newborns. Vitality at birth is often directly related to neonatal viability [2]. Vitality assessment is critical to identifying at-risk newborn animals and implementing interventions to prevent neonatal mortality. When considering pharmacological therapies, drugs such as caffeine [20,21] and naloxone have cardiorespiratory effects, benefiting physiological function. For energy supplementation, glucose administration is a common practice to supply newborns with the energy necessary to thermoregulate and move to the udder immediately after birth [22]. Oxygen administration to reduce or compensate for hypoxia states is another option for treatment. However, oxygen doses and their effect might differ between species, and one must consider individual cases according to the species and the newborn health at birth. Moreover, physical methods to promote vitality and reduce mortality rates, such as natural and artificial colostrum supplementation, provision of external heat sources, and controlled microclimates, are being studied to determine their effect on neonatal survival [23,24]. Nonetheless, because maternal and fetal factors alter neonatal vitality and the ability of the animal to adapt to the extrauterine environment [20], it is essential to create a comprehensive intervention protocol for susceptible animals.

Therefore, newborns are susceptible to hypoglycemia, hypothermia, dehydration, metabolic acidosis, less vitality, and death. The aim of this manuscript is to (1) understand the factors affecting vitality in farms animals (i.e., hypothermia, hypoxia, birth weight, glycogen depletion, neurodevelopment, and dystocia) and (2) review and analyze pharmacological and physical methods used to increase vitality in newborn farm animals.

## 2. Factors Affecting the Vitality of the Newborn

Significant impact on newborn vitality can have various factors, such as environmental (extrinsic), the environment in which the newborn is born and raised. Factors, such as adverse weather conditions for outdoor species (sheep, goat) or temperature, humidity, ventilation, and cleanliness for indoor species, can affect the health and vitality of the newborn. Management practices used on the farm can also affect the vitality of newborns. Proper nutrition, sanitation, and veterinary care can help ensure that newborns are healthy and vigorous. Other factors (intrinsic) are attributable to the fetus/newborn, such as hypothermia, hypoxia, birth weight, and glycogen depletion, or to neurodevelopment, related to other phenotypes, such as litter size, birth weight [25,26,27,28], and variation in birthweight. Others are attributable to the mother, i.e., dystocia, maternal ability, the absence of milk, the parity number, gestational age/length of gestation, the number of teats to nurse the newborn [25], and clinical factors (infectious, hypertensive, and metabolic disorders) and the general health status (notably nutritional) [29]. In the case of this review, we will only focus on the intrinsic factors, which will be discussed below.

### 2.1. Hypothermia

In all endothermal animals, modulation of thermoregulation is closely related to the stability of various cardiovascular, respiratory, renal, endocrine, nervous, muscular, and cellular functions [30], as well as environmental conditions that are not in the scope of this review. One of the leading causes of neonatal mortality in farm animals (i.e., lambs, calves, foals, piglets) and other mammals is hypothermia caused by a significant loss of heat or inhibition of thermoregulation, resulting from starvation when the offspring is unable to suckle [31]. Another cause of hypothermia is physiological immaturity coupled with the fact that when being expulsed from the uterus, the newborn’s temperature can drop up to 3.5 °C [32,33]. This physiological change can trigger hypoglycemia, hypoalbuminemia, growth retardation, or changes in the acid–base ratio, leading to multiorgan failure [34]. For example, pre-weaning mortality in piglets in EU countries remains around 15%, with some as high as 25% [35], with many due to “crushing” by the sow, an outcome often associated with postnatal hypoglycemia and hypothermia [36,37]. In all endothermal animals, modulation of thermoregulation is closely related to the stability of various cardiovascular, respiratory, renal, endocrine, nervous, muscular, and cellular functions [30]. One of the leading causes of neonatal mortality in farm animals (i.e., lambs, calves, foals, piglets) and other mammals is hypothermia, produced by a significant loss of heat or the inhibition of thermoregulation and heat production, resulting from starvation when the offspring is unable to suckle [31].

However, how does a neonate fall into hypothermia? The sudden drop in body temperature at birth reduces vigor and affects the newborn’s feeding ability. Consequently, colostrum intake (the only source of immunoglobulins, nutrients, and energy) to fuel for thermogenesis is diminished [38,39,40,41,42]. In addition, factors associated with the activation of the hypothalamic–pituitary–adrenal (HPA) axis [43] increase the concentrations of catecholamines and circulating cortisol, both in the fetus and in the neonates [44], generating changes in blood flow that can compromise the ability to thermoregulate [45].

Newborns use various mechanisms to minimize or compensate for hypothermia: shivering, vasomotor control, and the presence of brown adipose tissue (BAT) as an energy source (discussed in Section 3). However, these mechanisms can require much energy and thus perpetuate the problem. Mechanisms include the presence of thermogenic cells, such as BAT; the presence of hair; thickness of the dermis; body-to-mass ratio; behaviors at birth; locomotor abilities; and organ development [46,47]. In farm animals, the characteristics mentioned above can differ markedly between altricial and precocial species [22]. In many aspects, thermoregulation in altricial species is often further complicated due to the absence or shortage of hair, high body surface-to-mass ratio, and scant adipose tissue [48]. In contrast, in both species, the fact that newborns are born wet with amniotic fluid makes them susceptible to evaporative heat losses [49].

In piglets, shivering is a compensatory mechanism often observed, but with high energy demand. As a result, piglets have increased myofibril mass, blood supply, and triad proliferation (fat intake, maturation of adipocytes from multi to unilocular, improved thermal homeostasis) to improve shivering efficiently [39,50]. The case of newborn piglets differs from most mammals; they have almost no BAT, and their thermoregulation highly depends on shivering to produce heat. In addition, the sow does not lick the piglets to remove the blood and amniotic liquids and placenta fragments, meaning that the piglets are wet during some hours and have more risk of hypothermia, a particularity of the species. That is why, in newborn piglets, various molecular, ultrastructural, biochemical, and physiological adjustments are involved in the maturation of the energy metabolism of the musculoskeletal system at birth [39]. Nonetheless, hypothermia can also affect precocial species, such as ruminants. Despite having a better degree of neurodevelopment at birth, hypothermia in ruminants is a factor occasionally associated with mortality in newborns due to the drastic temperature change when exposed to the extrauterine environment in the first hours after birth [51].

### 2.2. Hypoxia

**Figure 1:**
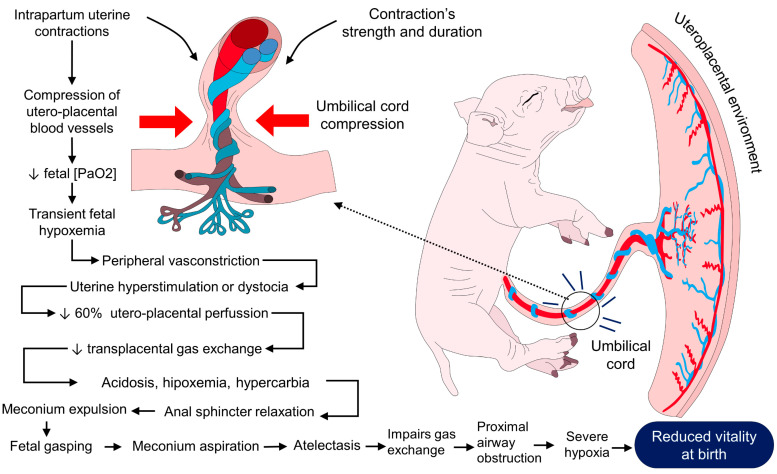
Hypoxia is a factor that reduces vitality. During intrapartum uterine contractions, uteroplacental blood vessels and the umbilical cord are compressed. Due to this compression, partial fetal pressure of arterial oxygen (PaO_2_) is reduced, causing transient hypoxemia in the fetus. When uterine contractions increase or uterine hyperstimulation occurs, around 60% of the uteroplacental blood circulation is reduced, affecting transplacental gas exchange and fetal oxygen supplementation. Along with hypoxemia, other physiological events, such as acidosis, hypercarbia, and meconium aspiration, can also be present, reducing a newborn’s vitality.

All factors that reduce the oxygen supply passing from the mother to the fetus promote hypoxia, i.e., constriction of uterine blood vessels, placentitis, and umbilical cord occlusion, among others [3]. Perinatal decrease in oxygenation through the umbilical cord leads to fetus asphyxia due to inadequate respiratory gas exchange, triggering metabolic acidosis [52]. The severity of perinatal hypoxia can be assessed at birth using various indicators, such as blood pH, CO_2_, and blood oxygen concentration, as well as various cardiopulmonary and neurological function tests [53,54]. Pre- and intrapartum uterine contractions can cause varying degrees of hypoxia. When suffering from perinatal stress, the body releases catecholamines, significantly impacting the activated metabolism, glycolysis, and hyperglycemia [36,54]. By increasing the metabolism, oxygen requirements will be higher [55]. Uncontrolled hypoxia can lead to asphyxia, and when this occurs intrapartum, the fetal lungs decrease the amount of surfactant, inducing respiratory acidosis [7]. In other words, when a fetus suffers from hypoxia (caused by umbilical cord or fetal head compressions [3]), there are some changes in fetal circulation, which increase intestinal peristalsis, causing relaxation of the tone of the anal sphincter, allowing meconium to pass into the amniotic sac [53]. In an attempt to survive, the hypoxic fetus initiates forceful respiratory movements with the glottis open, inhaling meconium-contaminated amniotic fluid, which is directed deep into the lungs. In turn, inhaled meconium produces obstruction and inflammation of the airways, translating into inadequate oxygenation and causing alveolar surfactant degradation [54], which is associated with asphyxia and pulmonary hypertension [55]. In a study by Bochenek et al. [56], piglet alterations during the hypoxic period demonstrated clinical signs consistent with encephalopathy. The clinical signs derived from peripartum hypoxia and pulmonary anomalies constitute Meconium Aspiration Syndrome (MAS). MAS defines the respiratory distress in newborns born during labor complicated by meconium-stained amniotic fluid [48]. According to Martínez-Burnes et al. [57], functional and structural repercussions of MAS include airway obstruction, atelectasis, chemical pneumonitis, hypoxemia, acidosis, pulmonary hypertension, and, in some cases, death (Figure 1) [58,59,60,61,62].

On the other hand, anemia in newborn piglets is common and is more frequently observed in large litters [63,64]. This is important because hemoglobin serves as an important non-bicarbonate, essential for oxygen transport [65]. Then, the hypoxic events that the newborn faces can cause a decrease in its vigor since the lack of oxygen can lead to the presentation of MAS. Therefore, one of the first therapeutic lines could be to try to improve the availability of oxygen to reduce the impact of this phenomenon.

### 2.3. Birth Weight

Birth weight in any species can significantly predict newborn survival [66]. There is controversy on this issue, and some authors point out that low birth weight can be compensated in the weaning period [67], while others point out that low birth weight can cause growth difficulties [68].

**Figure 2:**
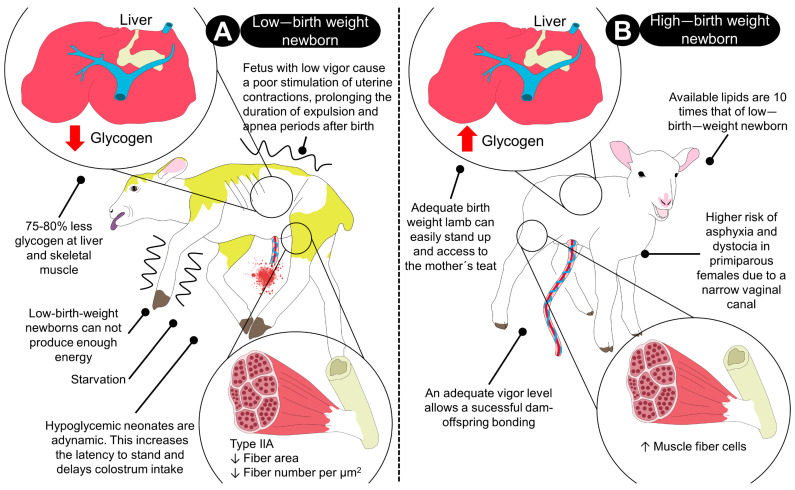
Importance of birth weight in the percentage of newborn survival. (**A**) In newborns with low birth weight, there are many limitations to being able to produce energy from their glycogen reserves in the liver and skeletal muscle because these are reduced between 75 and 80%. Cori’s cycle cannot be activated correctly because muscle fibers are reduced per µm^2^, and the lamb cannot get up and look for the mother’s teat, so it may starve to death. Low birth weight is closely associated with fetal and neonatal morbidity, inhibited growth and cognitive development, and chronic diseases. During oxygen deprivation in the event of fetal hypoxia–ischemia, compensatory mechanisms are responsible for redistributing cardiac output, centralizing blood flow to vital organs, and reducing oxygen consumption. Additionally, an increase in peristalsis with expulsion and staining of meconium on the skin can be present, as illustrated in the lamb on the left side. (**B**) In contrast, high-birth-weight newborns have adequate vigor, can stand up immediately to consume colostrum, and have enough energy reserves. These reserves help to achieve thermoneutrality, together with the higher amount of muscle fibers, and enough hepatic glycogen reserves. In addition, the lipid reserves in lambs born with normal weights is 10× the amount of available lipids in those that have low birth weight. Although normal-weight newborns have increased chances to survive, when the female has a narrow birth canal, these animals can experience dystocia and a prolonged expulsion period that might predispose them to meconium aspiration. Type IIA: muscular fibers type IIA.

Two biological factors could influence neonatal viability in farm animals: birth weight and nutritional status in the perinatal period. Diverse studies suggest that there is a negative association between low birth weight and survival during this period [39,69,70,71], and it has been demonstrated that a positive correlation exists between the ability to raise thermostability and a newborn’s weight. Nonetheless, negative aspects can also be found in newborns with normal weight born from nulliparous females with narrow birth canals (Figure 2) [72,73,74,75].

In the same way, there have been studies carried out in sheep [76,77], pigs [36,78], and cows [79,80] that have shown how factors, such as the dam’s weight and body condition, parity, newborn’s weight at birth, breed and age of the mother [47,81], number of siblings in the litter, and maternal and offspring behavior, can influence the survival of the newborn [82]. It is often assumed that low birth weight in newborns is usually associated with prematurity, placental insufficiency (i.e., newborns, piglets, lambs), or maternal undernutrition (i.e., lambs) [83]. In piglets, one of the possible reasons for the lower viability and low birth weight can be immaturity [84].

According to Schmidek et al. [80], reduced birth weight during normal calving may also be associated with poor vitality. They suggest that up to 74% of calves with reduced vigor die before weaning. The availability of liver glycogen and skeletal muscle is decreased by 70–85% in newborns with low birth weight. In the same way, the amount of available lipids in low-birth-weight animals (i.e., pigs, lamb) is only about 10% of the amount available for those born with adequate weight, and these factors can affect the ability of the newborn to generate the energy necessary to stand up or access the mother’s teat and suck colostrum [83].

### 2.4. Glycogen Depletion

In piglets, the risk of hypoglycemia is associated with sibling competition to get better or functional teats, resulting in high colostrum intake variability among piglets [55,84]. The colostrum requirement for newborn piglets is 180–250 g [85], but on some occasions, these requirements are not covered, especially in sows rearing large litters. Animals not reaching their colostrum intake requirements have to rely on using glycogen, which can be inappropriate for weak newborns [86]. Glycogen reserves are stored in the liver and the muscle and are usable by the newborn from 12 to 24 h and 24 to 36 h after birth, respectively [55]. In any case, the liver’s glycogen reserves are depleted in most species from 12 h after birth. Then, the newborn depends on the energy supply obtained through colostrum or gluconeogenesis. However, it is important to mention that gluconeogenesis occurs in the newborn’s liver simultaneously with the appearance of phosphoenolpyruvate carboxykinase after the rise of plasma glucagon and the fall of plasma insulin [87]. This synthesis of energy requires that activation of the Cori cycle take place, through which the production of lactate in muscle cells arises, its transport to the liver, its conversion into glucose by gluconeogenesis, and its return to the muscle to be converted back into lactate ensuring the functioning of the muscles in periods of great activity, such as shivering. The Cori cycle involves glucose consumption in muscle under anaerobic conditions, producing lactate from pyruvate and nicotinamide adenine dinucleotide (NADH) synthesized during glycolysis, with a considerable expenditure of energy at the liver [88]. The problem with activating this cycle is that lactic acidosis occurs in the muscles, which can decrease the blood’s buffer system efficiency, leading to physical fatigue caused by oxygen debt [83]. Moreover, it is a cycle that costs 6 adenosine triphosphate (ATP) in the liver, and it is a cycle that cannot continue indefinitely [6,89].

By depleting glycogen reserves and thus generating hypoglycemia, the newborn is led to a decrease in heat production that causes hypothermia and compromises brain function, which can lead to a coma or even seizures and death [83]. The fact that the animal at birth has limited energy reserves could directly impact its vitality. Therefore, therapeutic strategies should be focused on promoting the vigor of the newborn.

### 2.5. Neurodevelopment

According to Mellor [90], newborn mammals can be classified according to their neurological maturity in (1) neurologically mature newborns (offspring of guinea pigs, primates, and ungulates, such as deer, cattle, horses, pigs, goats, and sheep); (2) neurologically moderately immature newborns (offspring of cats, dogs, ferrets, bears, rabbits, hamsters, mice, and rats); (3) neurologically exceptionally immature newborns of marsupials (wallabies, opossums, and kangaroos). Most farm animals are neurologically mature at birth, except rabbits, which are moderately mature at birth. In the neurologically mature group, the mother–young bond quickly establishes itself; in the moderately immature group, the female makes nests and burrows to protect her young; and in the exceptionally immature group, immediately after birth, the young hide in the pouch of the mother, where they remain safe and nursing [91,92].

Poor neurodevelopment in the newborn could possibly affect vitality because it could have a limited physiological response. In this sense, it has been reported that limited neurodevelopment in ruminants can decrease their thermoregulatory capacity and, therefore, the response to stimuli at extreme temperatures [93]. Thus, with limited responsiveness, it may fail to respond favorably to events, such as a drop in body temperature, by decreasing heat loss through changes in the superficial microvascular response due to hypothalamic activity, and the autonomic nervous system, which could affect vitality in the newborn [94]. Regarding their sensory capabilities, mature neurological newborns have the proprioception, musculoskeletal system, and vestibular function sufficiently developed to stand up immediately after birth; in the same way, a developed sense of smell and vision allow them to track and focus on objects [95,96]. Neurological moderately immature newborns have developed taste, smell, touch, proprioception, nociception, and thermal sensitivity; however, visual and hearing capacities are absent and not functional until cortical–subcortical connectivity is established at about 10–17 days after birth [97,98].

Intrauterine life gradually prepares the fetus for birth, and it develops the sense of smell and taste to recognize its mother’s scent and easily find the udder to consume colostrum. In the same way, the fetus develops essential neuroplastic responses, presenting some outlines of learning and memory that can be useful in extrauterine life [90]. Given the evidence, it is clear that the degree of neurodevelopment directly correlates with the degree of vitality in the newborn animal. Therefore, it is necessary to resort to therapeutic strategies to promote vitality in the different species.

### 2.6. Dystocia

Previously, we have mentioned that some maternal causes could interfere with the fetus’s and newborn’s vitality (dystocia, maternal ability, the absence of milk, parity number, or clinical factors). We will focus on dystocia because these maternal factors directly influence the peripartum period and can cause a severe circulatory imbalance, producing low oxygenation in the fetus and newborn [5].

Dystocia compromises fetal circulation, causing intrauterine fetal hypoxia that triggers a redistribution of blood from less sensitive organs, such as the intestine, to vital structures, such as the brain and the heart. Reduced intestinal perfusion promotes peristalsis and reduces the muscle tone of the anal sphincter, resulting in the passage of meconium into the amniotic sac [7], thus causing the MAS mentioned above and its pathophysiology previously described in the hypoxia section [99,100].

In cows, the degree of calving stress depends on several factors, such as calf size, presentation, the dam’s pelvic dimensions (correlated with age), the force of contractions, insufficient cervix dilation, and uterine torsion. These factors, alone or in combination, can trigger labor dystocia [101]. In the presence of dystocia, the cow may experience reduced milk production and/or uterine infections, leading to increased production costs and decreased fertility [79]. According to González-Lozano et al. [102,103], in swine production, dystocia increases the risk of piglets with prolonged latency to first udder contact, generating weakness and thereby decreasing their vitality scales.

Commonly, exogenous oxytocin is applied on the farm and domestic animals to reduce long or dystocia births. It has been seen that this could cause an increase in the number of stillbirths if it is not appropriately applied [62,104]. This is because myometrial contractions can cause a decrease in oxygen supply to the fetus, especially in polytocous species [12]; hence, induction protocols should be assessed to better suit newborns’ developmental needs [50]. Under all the above, it can be concluded that dystocia, the age of the dam, and birth weight have the most significant impact on newborn vitality and, in the long term, on neonatal survival.

## 3. Physiological Mechanisms of the Newborn to Achieve Thermostability and Improve Vitality

One of the most adaptive changes the fetus makes to compensate for thermal variations in the perinatal period include the reorganization of the muscular circulation [29] of the hindlimbs and its return to the brain and heart and reduced movements of the limbs or body. After the onset of respiration, the increase in peripheral blood flow in response to the elevation of blood PO_2_ releases lactic acid produced in the muscle by anaerobic metabolism. Therefore, transient lactacidemia after the onset of respiration can be expected in normal parturition [105]. Among the relevant hormones in the secretion of pulmonary fluid, we can mention catecholamines, thyroid hormones, and cortisol elevation [6].

### 3.1. Brown Adipose Tissue

Non-shivering thermogenesis is the production of heat without shivering. This occurs when the neonate’s brown adipose tissue (BAT) is activated and produces heat by burning stored fat (unlike white adipose tissue, primarily used for energy storage). BAT gets its name from its dark color, which is caused by numerous mitochondria. These mitochondria contain a protein called thermogenin, also known as uncoupling protein 1 (UCP1), which uncouples the electron transport chain from ATP synthesis, allowing the energy emitted by burning fat to be released as heat instead of being used to produce ATP [106]. The type of species highly influences the degree of thermoregulatory neurodevelopment. Ruminants, classified as precocial, can maintain a constant body temperature during the early postnatal period, even in cold environments [107]. In these species, non-shivering thermogenesis is the most used mechanism by neonates. For example, in lambs (*Ovis aries*), about half of the cold-induced metabolic peak comes from non-shivering thermogenesis. Therefore, metabolic-active BAT during the early postnatal period is essential [108].

Thermogenesis by BAT is crucial in the neonate of most species and represents the first energy resource available during the postnatal period. In response to cold, sympathetic stimulation dramatically increases lipolysis and blood flow through the fat depots to provide direct heat [109].

**Figure 3:**
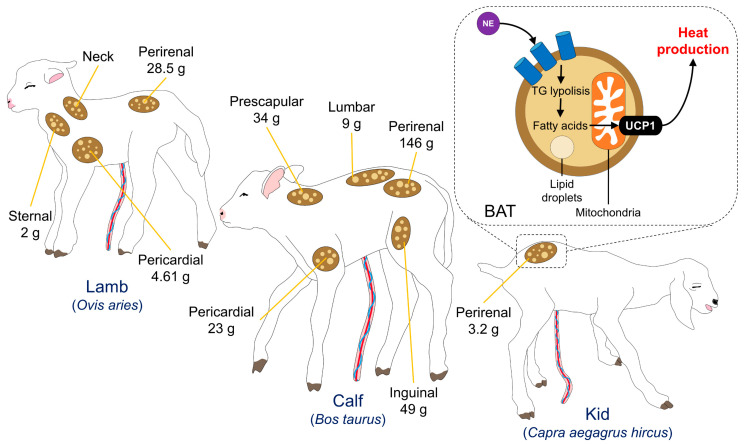
Amount and distribution of brown adipose tissue (BAT) in lambs, calves, and kid goats. According to the species, different levels of BAT are available at birth. For example, concerning the animal’s weight at birth, newborn lambs have less pericardial BAT (4.61 g) than calves after birth (23 g). Similarly, perirenal adipose tissue is higher in calves (146 g vs. 28.5 g) and the only reported source in kids, which have 3.2 g of BAT. These values influence the ability of newborns to thermoregulate and prevent critical heat losses. NE: norepinephrine; TG: triglycerides; UCP1: uncoupling protein 1.

The quantity of body fat in the newborn varies markedly between species. Precocial animals, such as lambs and calves, are born with well-developed BAT reserves that quickly atrophy and are replaced by white adipocytes shortly after birth [109,110]. In contrast, altricial species are born with zero or minimal amounts of BAT, whose recruitment increases when exposed to low temperatures during the first weeks of birth [109]. BAT replacement or atrophy after birth depends on the species: it disappears in rabbits, sheep/cattle, and goats within a month, from 2 to 3 days, and 2 to 6 days after birth, respectively [109]. BAT is high in rabbits, but there are small amounts in sheep, cattle, and horses, while it is almost non-existent in newborn pigs [105]. Nonetheless, adipose tissue represents only 2% of the body weight of livestock species, and is distributed in the prescapular, inguinal, and prerenal regions (Figure 3) [110,111,112]. However, in species with limited energy reserves, at birth (such as piglets), colostrum intake and other mechanisms to preserve heat, such as shivering, vasomotor control, and postural behavioral changes, are critical to prevent hypothermia [113]. That is why, in newborn piglets, various molecular, ultrastructural, biochemical, and physiological adjustments are involved in the maturation of the energy metabolism of the musculoskeletal system at birth [39]. Consequently, BAT thermogenesis is essential in the neonate of most species and represents the predominant resort to use during the postnatal period. Nevertheless, colostrum consumption is crucial in species with limited energy reserves to counteract hypothermia.

### 3.2. Shivering

Shivering is a thermogenic mechanism of repetitive and rapid skeletal muscle contractions when the body is exposed to cold weather or a fever. One of the main differences between immature and mature species is their morphological characteristics, such as the percentage of body fat, the surface–volume ratio, and the adenosine triphosphate (ATP) necessary to maintain contractions; this could influence the intensity of the shivering [114]. The body core temperature of mammals is regulated by the central nervous system, in which the hypothalamus’s preoptic area (POA) plays a pivotal role [115]. Shivering is regulated by structures that connect the POA with the parabrachial nucleus, the dorsomedial hypothalamus, the raphe pallidus, and spinal cord motor neurons. Although it employs thermosensitive neurons in the POA and spinal cord, these can also be activated in metabolic thermogenesis through BAT [116].

The shivering process uses the oxidation of carbohydrates, lipids, and proteins from muscles’ reserves and circulating blood [114]. It can increase oxygen consumption 20 times, increasing the aerobic activity of muscle fibers and leading to the oxidation of fatty acids [117].

Although shivering is the most efficient mechanism to produce heat and achieve thermal balance in precocial species, such as ruminants, during exposure to cold [118], it cannot be used as a primary thermogenesis method due to the immaturity of muscle tissue in ruminants [107], especially in lambs of the Merino breed [119]. Thermogenesis through shivering is an efficient method for compensating for hypothermia in the newborn, which helps to complement the metabolic response; however, it can quickly deplete energy reserves.

### 3.3. Vasomotor Control

Studies in a wide range of species show that the set-point of the major hormonal systems that mediate the stress response (including the autonomic nervous system and hypothalamic–pituitary–adrenal (HPA) axis) can be altered during early life [120]. Vasomotor changes depend on the activation of the sympathetic system [121]. In the case of cold stimuli, the participation of the HPA axis, the secretion of catecholamines (epinephrine and norepinephrine), and their action on the receptors located in the blood vessels generate a vasoconstriction effect [122,123]. The objective of vasoconstriction is to redirect the blood flow of the extremities or peripheral structures toward internal organs and vital centers, limiting heat loss through the skin [124,125,126].

### 3.4. Postural and Behavioral Changes

Many postural changes in newborns aim to avoid heat loss and/or provide additional warmth. For instance, it is observed in pigs that, to reduce heat loss at birth, they adopt positions such as snuggling with littermates [127], or they lay in the sternal position to reduce the contact surface with the ground and prevent heat loss [128]. Due to the direct effect of behavioral changes on the newborn’s thermoregulatory capacity, they also impact the animal’s vitality. For example, in ruminants, it is reported that the average time to stand up is 20–22 min, and to suckle later is 50 min. If this time is longer, the animal may have weakness due to insufficient energy consumption. This could be related to the lack of availability of energy resources, which would perpetuate the difficulty in standing up and suckling [129]. In a study of rabbits *(Oryctolagus cuniculus)* exposed to thermal challenge with cold, it was observed that they had a greater incidence of presenting the behavior of huddling, and lower levels of triglycerides and BAT was also found in them compared to control animals [130]. Although this demonstrates that changes in behavior and posture are compensation mechanisms against a factor that affects vitality, they could also be indirect indicators of energy resource levels. For this reason, once these signs of weakness have been recognized, it is necessary to adopt therapeutic resources to promote vitality in the newborn.

In altricial species, behaviors such as crowding, seeking warmer sites, and calling to the mother are more commonly observed [131]. In the case of the rabbits *(Oryctolagus cuniculus*), they frequently adopt behaviors such as snuggling, rooting, and climbing, in addition to maintaining close contact with the rest of the litter to achieve a better position within the nest; moreover, this ensures a source of heat and food [132]. Through these physiological and behavioral strategies, newborns can increase their vitality and thus achieve survival.

## 4. Therapies and Methods Applied in Neonates to Promote Vitality

### 4.1. Pharmacologic Therapies

#### 4.1.1. Energetic Supplements: Dextrose and Colostrum

The vitality and thermoregulatory capacity of the newborn is mainly determined by its energetic reserves, which are needed for thermogenesis [51,128]. For this reason, one suggested therapy is using energy supplements that provide calories to the newborn to achieve thermal stability [22]. Low blood glucose availability is recognized as one of the risk factors for newborn mortality. For example, McCauley [133] suggests that piglets with low birth weight can have low vitality and greater susceptibility to hypothermia because these animals have fewer energy resources to compensate for their heat loss, which has also been verified in ruminants [134].

In ruminants, the intravenous (IV) administration of 5% dextrose solutions at a rate of 500 mg/kg^−1^ has been suggested as a therapy that reestablishes blood glucose levels when they are lower than 60 mg/dL [135]. In this sense, Eales et al. [136] reported that intraperitoneal administration of 10 mL/kg of 20% glucose and rewarming with air at 40 °C improved survival in lambs. This suggests that providing energy resources to the neonate could increase its vitality and thermoregulatory capacity. However, controversies have arisen with pharmacological treatments. Oral administration of 40% dextrose in lambs increased the chance of their survival 3 h after birth, but was not superior to the implementation of a physical warming technique [137]. There is controversy regarding whether it is more effective to treat hypothermia or hypoglycemia in a newborn with low vitality.

Precisely, Engelsman et al. [138] evaluated the effect on the rectal temperature (RT) and glucose levels of administering energy supplements (glucose or colostrum) in three sessions. They evaluated 88 piglets receiving subcutaneous glucose (50 mg/kg) or 20 mL of colostrum at 35 °C, or a combination of both. They found no difference in RT among the three treatments, but the combined use of glucose and colostrum resulted in higher blood glucose levels. This suggests that high-quality energy resources, like colostrum, are related to vitality. Previously, Dividich and Noblet [139,140] mentioned that promoting colostrum consumption is an alternative to synthetic pharmacological therapies because it increases energy reserves by at least 30% in piglets.

The increased availability of energy resources is not the only benefit of this treatment. According to Silva et al. [141], the thermoregulatory response of newborn Holstein calves is altered when fed different volumes of colostrum (10%, 15%, and 20% of their weight). They found that the animals that consumed 15% and 20% colostrum had a higher pre-scapular temperature than those that consumed 10%; in addition, the total leukocyte counts increased. Both benefits could be associated with increased vitality because the thermoregulatory and immunological capacity would improve newborn survival. Colostrum contains different cell growth factors that promote differentiation, such as insulin-like growth factors (TGF1 and IGF-2), transforming growth factor b (TGF-b1 and TGF-b2), growth hormone, epidermal growth factor, and insulin [142,143]. Similarly, Muns et al. [144,145] observed an increase in IgG levels at days 4 and 5 of life in piglets orally supplemented with colostrum after birth.

Due to the presence of these elements, colostrum could reduce the incidence of diseases in newborns. Therefore, energy supplementation is a viable therapeutic alternative to increase vigor in weak animals with signs of hypothermia.

#### 4.1.2. Caffeine

**Figure 4:**
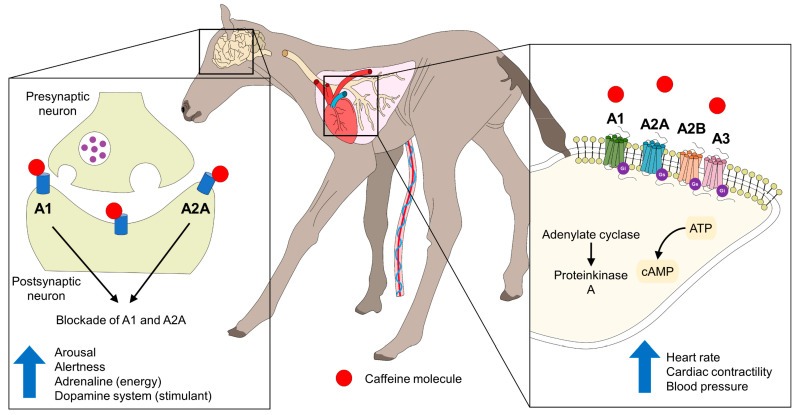
Cardiorespiratory and neural effects of caffeine. The mechanism of action of caffeine, binding to adenosine receptors (A1, A2A, A2B, and A3) in the cell membrane, causes cardiac responses that can improve a newborn’s vitality by increasing heart rate, cardiac contractility, and blood pressure. Moreover, it acts on postsynaptic neurons and in the blockade of A1 and A2A. Binding these receptors increases arousal and alertness by accumulating adrenaline and activating the dopaminergic system. ATP: adenosine triphosphate; cAMP: cyclic adenosine monophosphate.

Caffeine is a methylxanthine antagonist of adenosine receptors in the Central Nervous System. Caffeine acts as a stimulant on physiological variables, such as the heart and respiratory rates (Figure 4) [20,21,146,147]. This drug has been used intravenously, orally, or subcutaneously at 20 mg/kg to promote vitality, increase oxygenation, and reduce metabolic changes due to perinatal asphyxia in farm animals [148].

Some studies have suggested that caffeine administration between 20 and 30 mg/kg 8 h after the birth of piglets with high weight and diagnosed with asphyxia presented a positive response, increasing body weight by 19% at weaning [149]. However, it may have the opposite effect when administered to animals with low weight and lower vitality, as a lower colostrum intake is usually accompanied by lower weight gain [150]. These authors suggested that caffeine as the only therapeutic intervention would have little effect, as was observed with the additional use of glucose. In addition, Robertson et al. [151] evaluated the effect of caffeine administration at 20 mg/kg to improve Merino lambs’ vitality. They observed that the lamb mortality was similar in both the control and the treated group, suggesting a nonsignificant effect on vitality.

A possible method to improve caffeine’s effect would be combining energy supplements, as suggested by Jarrat et al. [152], who evaluated the effect of caffeine and glucose supplementation in 398 piglets at birth. Oral administration was randomly proportioned with 30 mg of glucose, 30 mg of caffeine, and a combination. This treatment did not benefit animals with ideal or high weight, but improved growth between days 1 and 3 by 0.9 kg in animals with low birth weight. These results suggest that the caffeine administration increased the energy capacity so that the animal could remain stable during the critical period. However, the timing and concentration of caffeine might determine its effect. For example, a study evaluated the optimal concentration and duration of caffeine in Merino ewes at 120 and 140 days of gestation. In animals receiving 10 or 20 mg/kg of caffeine in the feed at 120 days of gestation and 20 mg/kg after 140 days of gestation, it was observed that the high administration of caffeine from day 120 allowed the lambs to present a higher rectal temperature, greater suckling attempts, and a longer sucking time [153]. This suggests that caffeine administration during late pregnancy would be a more viable option to guarantee its effectiveness in the newborn.

Contrarily, in premature lambs, it was observed that caffeine administration did not affect the carotid flow, heart rate, and oxygen saturation [154]. This is similar to what was reported by Menozzi et al. [155], who carried out a pharmacokinetic study of the administration of oral caffeine in sows and found that 24 h after its administration, plasmatic concentrations of 13.77 ± 0.97 mg/mL were shown.

The literature shows that caffeine administration can be used before birth to improve the vitality and respiratory capacity of newborns. However, more than administration of caffeine alone is needed to achieve this effect, and the combined use of energy supplements would help to achieve better results.

#### 4.1.3. Naloxone

Naloxone is an opioid receptor antagonist that reduces the physiological effect of endogenous opioids, such as dynorphin, enkephalin, and endorphins, at the brain level [156]. The physiological effect of these substances is the decrease in the respiratory rate and tidal volume in the body because the expression of opioid receptors in the respiratory center of the cerebral cortex, thalamus, and baroreceptors in the carotid bodies has become evident [157]. The antagonism of these receptors likely decreases this effect and increases the ventilatory capacity. The therapeutic effect of naloxone has been previously described in newborn animals. For example, Hazinski et al. [158] evaluated the effect of naloxone administration at 4 mg/kg in 19 rabbit pups. They observed that the minute volume increased by 50 mL and the tidal volume increased by 8% in animals administered naloxone compared to the baseline event. In addition, blood gas analysis showed a 5 mmHg decrease in CO_2_ levels and a 2 mmHg increase in pO_2_. These authors concluded that endogenous opioids participate in respiratory control; therefore, their antagonism can positively intervene in respiratory activity. Interestingly, in a study carried out in paralyzed and vagotomized piglets, the administration of naloxone caused an increase in phrenic neural activity, which increased the respiratory output by 122 ± 36% and also the output per minute by 54 ± 12% [159]. This could demonstrate that naloxone has a beneficial effect on the respiratory capacity of the newborn with signs of low vitality or asphyxia. However, the results are limited in whether this could be related to improving thermoregulatory capacity and whether these effects can be prolonged.

#### 4.1.4. Oxygen Therapy

Oxygen is essential for aerobic respiration, yet is potentially toxic, causing what is described as oxidative stress when there is an imbalance between the reactive oxygen species produced by aerobic respiration and the body’s ability to detoxify these products. The capability to efficiently deliver sufficient oxygen to the tissues for optimal cellular function while minimizing oxidant-induced tissue damage has been achieved through complex physiological processes developed through evolution [160]. Oxygen therapy has been suggested as an alternative therapy to improve the vitality of newborn animals due to the characteristic of perinatal asphyxia caused by meconium aspiration [61]. For this reason, it has been suggested that the administration of 100% oxygen could counteract the effects of perinatal asphyxia and consequently improve vitality in the newborn [161]. In fact, due to asphyxia, cerebral hypoxia can also become evident due to the decrease in blood oxygen and local hemodynamic changes that would cause the demand for this available resource to be lower [162].

Intranasal oxygen support has been suggested as a form of treatment for perinatal asphyxia. For example, Bleul et al. [163] evaluated the effect of intranasal oxygen administration on blood gas variables in 20 newborn calves with respiratory distress syndrome. They found that this treatment significantly increased the partial pressure of oxygen (PaO_2_) by 20.3 ± 8.8 mmHg and SaO_2_ by 10% within the first 12 h after birth, which increased the survival rate (treatment group = 9/10 vs. control group = 4/10). This suggests that this simple treatment would be an option to improve blood variables and vitality in the newborn. In fact, in humans, it has been found that the implementation of this therapy, apart from improving the fraction of inspired oxygen, decreases acid–base changes and oxidative stress during neonatal resuscitation due to respiratory stress, so it could be considered a method to counteract the acidosis caused by this event [164].

The possible explanation for this process could be that during birth, there is decreased secretion of the surfactant substance, producing pulmonary atelectasis, which would hinder gas exchange and increase hypoxemia in the individual [165]. Therefore, for improving gas exchange in the newborn, it is necessary to consider that there are also processes of pulmonary hypertension and an increased airway [166]. Thus, a ventilation maneuver would increase the possibility of gas exchange due to the gas pressure that would overcome the increase in airway pressure.

Therefore, oxygen therapy helps increase the newborn’s limited energy resources due to asphyxia during this process. In addition, it increases the ventilatory reserve that can be diminished by the aspirated meconium. However, it is necessary to understand that there may be an increase in airway pressure, which would hinder gas exchange, so ventilation maneuvers would help to increase this process.

### 4.2. Physical Methods

#### 4.2.1. Colostrum: Natural and Artificial Supplementation

Providing good-quality colostrum to newborns is a practice that has improved in the last 20 years, reducing the incidence of insufficient colostrum intake in several species. For example, supplementation has reduced insufficient colostrum intake in dairy calves to 19% in dairy farms [167]. Passive transfer of immunoglobulins (Ig) through the colostrum and the methods to ensure an adequate immune transfer are relevant because they are related to the mortality rate and disease susceptibility [168,169].

Large animals (e.g., ruminants, foals, and piglets) are born agammaglobulinemic due to the placenta structure that cannot pass maternal Ig to the fetus. Therefore, passive immune transfer depends on the intake of Ig through colostrum [170,171,172]. Colostrum deficiency is associated with increased morbidity and mortality in newborns. In the case of dairy calves, if they consume less than 10 g/L of colostrum, they could have an increased susceptibility to infectious diseases [171].

It is important to ensure colostrum intake from the mother immediately after birth—or provide colostrum from another dam going into parturition or even frozen colostrum—because, for example, in foals, their colostrum absorption efficiency is reduced quickly, reaching only 1/5 of its efficiency at 3 h after birth [23]. In the case of Jersey calves, high-quality colostrum fed immediately after birth and after 12 h resulted in high concentrations of IgG (45.66 mg/mL) [169]. During the first hours of life, it is crucial to consume adequate amounts of colostrum, as reported in piglets, in which the serum IgG concentration affects the survival of animals. In contrast, newborns with less than 1000 mg/dL serum IgG had only a 67% chance of survival during the first 72 h after birth [173]. In the Bragg et al. [157] study, factors associated with the likelihood of having low IgG concentrations at birth in dairy calves were colostrum intake using artificial bottle/tube feeding systems and calving assistance. Therefore, because newborns are not usually considered immunocompetent until reaching weaning ages, at birth, they are exposed to increased mortality rates due to their lack of immunocompetence [174].

To enhance immunocompetence in newborns, an adequate immune transfer and amount of Ig are necessary to improve their vitality. Under this connection, supplying colostrum in the newborn could improve the immune capacity to face infectious events or those that require high energy consumption. There are different methods to provide colostrum, whether from the biological mother, frozen colostrum, other species, and commercial supplements. In Canary goat kids, the administration of refrigerated and frozen colostrum (5% of the kid’s body weight) resulted in higher IgG concentrations (25.47 ± 19.89 mg/mL and 15.84 ± 5.91 mg/mL) at 24 h and 36 h post parturition, when compared to commercial sheep colostrum. Additionally, birth weight was related to IgG values, where kids weighing less than 2.5 kg had lower IgG concentrations [170]. In the same species, serum IgG concentrations and daily weight gain did not significantly differ between kid goats receiving natural colostrum and a cow supplement before ingesting the mother’s colostrum. However, there was a mortality rate of 4% for the individuals in the supplement group, suggesting that supplementing the mother’s colostrum does not benefit the newborn [175].

Contrarily, providing 10 g of IgG in 70 g of colostrum powder in 1-day-old Holstein calves resulted in less diarrhea (6.1% vs. 9.7%) and required fewer antimicrobial treatments than control animals. Although the mortality in the supplemented group was 7.7%, and 26.1% in the control animals, the authors reported no significant influence of colostrum supplementation on mortality and the incidence of respiratory diseases [176]. A study regarding colostrum in standard, thoroughbred, Arabian, and warm-blood foals reported no differences between normal foaling and dystocia. However, the serum IgG concentration at 24 and 48 h was lower in dystocia cases [177]. Therefore, knowing the parturition process in domestic animals is important when considering colostrum supplementation therapies. Supplementation in foals is recommended when the newborn is born from a mare with poor-quality colostrum (less than 20% BRIX). Although the amount of supplemented colostrum might vary, around 500 and 1000 mL is recommended [178]. One common practice is to provide colostrum replacers to newborns. However, in Holstein Friesian calves, neonates fed a replacer had lower body mass and lower blood neutrophils and monocytes (0.08 and 0.06 × 10^9^/L, respectively), as well as lower total serum proteins (44.34 g/L) than calves consuming maternal bovine colostrum [179].

Another factor to consider when deciding to provide colostrum is parity. In the case of sows, it is an important factor that alters colostrum quality and IgG concentrations, which can be 5% higher in multiparous animals [173]. Hyper prolific sows with litters of up to 20 piglets prolong the farrowing and increase the competition for colostrum intake, 2 factors that affect the piglets’ vitality and immunocompetence [180]. Similar information has been reported in Holstein cows, where colostrum from multiparous cattle had higher Ig concentrations (around 26%) and total proteins [172]. Maciag et al. [174] evaluated the effect of piglets consuming colostrum from gilts and sows and those bottle-fed with a commercial milk formula during the first 24 h after farrowing. Piglets’ serum Ig concentration and lymphocyte proliferation were assessed at 24 h and at 20 days after birth. The authors found that piglets suckling natural colostrum had higher Ig concentrations (particularly from sows with approximately 103.27 ± 12.8 mg/mL of Ig concentrations) and greater ability to produce B and T cells. At the same time, animals fed the milk replacement presented diarrhea and had lower body weights and a higher mortality rate (8/14 piglets, 57%) [174]. Piglets allowed to suckle naturally, but supplemented with artificial colostrum twice a day resulted in body weight increases after week 1, daily weight, and higher IgG counts [181]. This suggests that letting the newborn suckle ad libitum from their mother and supplementing with artificial formulas provides performance benefits for the animals and, probably, improves their vitality. As mentioned by Uddin et al. [182], in a study with 140 piglets from 10 sows, animals with Apgar vitality scores close to 2—in a scale of 0 to 2 (average of 1.42 ± 0.07)—consumed higher amounts of colostrum (*p* < 0.05), were born earlier (order 1 to 5), and maintained higher body weight at birth and until weaning ages. It is important to provide these elements to piglets, since factors such as birth order and low colostrum intake are associated with asphyxiation during farrowing, a relevant issue that must be considered when trying to reduce piglet mortality in farms.

Besides the nutritional components of colostrum, in the case of foals, the known neonatal maladjustment syndrome is a disorder that can alter the suckling ability of the foal to consume colostrum. Authors such as Aleman et al. [183] reported that an alternative to address this issue and prevent the consequences of the disorder, namely, the “Madigan squeeze method” applied as pressure for 20 min, helps the newborn to regain alertness and be able to bond with the mare and stand up quickly to consume colostrum.

#### 4.2.2. Temperature Drops Immediately after Birth—Sources of External Heat and Drying

Consuming colostrum immediately after birth is also linked to newborns’ ability to maintain body temperature because it serves as a fuel for heat production and thermoregulation [77]. Assessing the rectal temperature (RT) in newborn animals is the gold standard to evaluate their thermal state and how the environment influences their body temperature. Piccione et al. [184] studied the maturation and temperature pattern during the first month of life in foals and lambs. The authors found that animals could maintain a constant high temperature within 10 days post-parturition. In lambs, RT increased from 38.1 °C after birth to 39.4 °C on the 24th day of life. In contrast, foals went from 37.4 °C to 38.3–38.7 °C at 25 days after foaling. In Scottish blackface and Suffolk lambs, RT was lower in animals with low birth weight and those Suffolk animals, showing a difference even between breeds. Animals with a longer latency to suckle (114 min) and ingest colostrum also had lower RT, which was maintained for the first 3 days after lambing [77]. Aleksiev et al. [185] also reported differences between breeds in Pleven black head, Bulgarian, and Bulgarian X East Friesian cross lambs. In all animals, during the first 12 h, there was a fall in RT; however, Bulgarian lambs recorded the lowest temperatures, which was significant at 1 h post-lambing (39.8 °C vs. 40–40.1 °C in the other breeds). In that study, twin lambs also had lower temperature values than single animals. In contrast, in 13 Maltese kid goats and 13 Comisana lambs, Giannetto et al. [186] reported that RT was higher in kids and twins than in singletons and could be due to the physiological maturation at birth. In both species, the RT decreased during the first 33 h post-birth.

**Figure 5:**
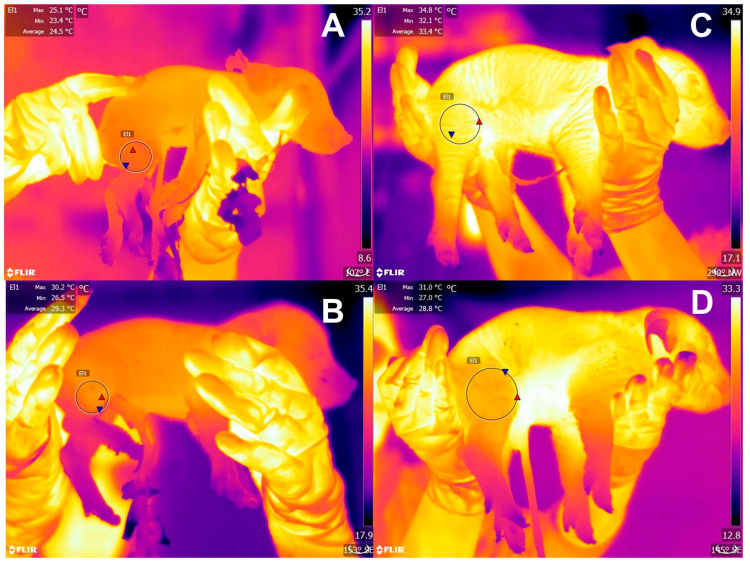
Thermal response of newborn piglets, evaluated by infrared thermography. Through infrared thermography and the delimitation of the region of interest in the pelvic limb (El1), factors such as weight and birth order can be assessed and associated with low birth temperatures, as shown in piglets from the same litter. (**A**) A piglet with a low birth weight (0.60 kg) and birth order eight had a minimum temperature of 23.4 °C. (**B**) A newborn weighing 0.62 kg and birth order five had a minimum temperature of 26.5 °C. In contrast, when comparing piglets from birth order one (**C**) and three (**D**) (weighing 1.58 and 1.63 kg, respectively), the minimum temperature of piglet (**C**) (32.1 °C) is 8.7 °C above piglet (**A**) and 5.6 °C above piglet (**B**). Minimum temperature of piglet (**D**) (27 °C) is maintained higher than (**A**,**B**). Red triangles mark the maximum temperature and blue triangles the minimum values in each thermal window.

**Figure 6:**
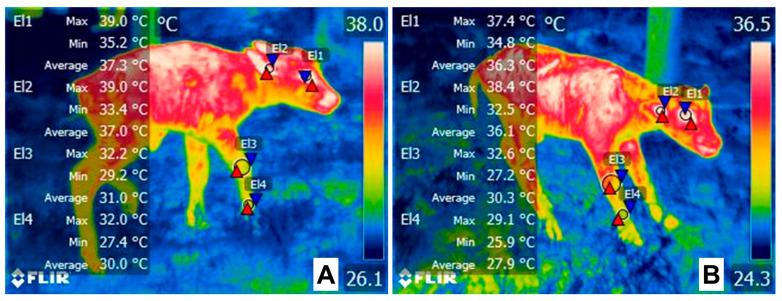
Comparison between central and peripheral thermal windows in newborn calves of water buffaloes with different thermal states. (**A**) Normothermic newborn. Central thermal windows, such as periocular (El1) and auricular (El2), have an average temperature of 37.3 °C and 37.0 °C, respectively. Peripheral regions considered in the elbow (El3) and metacarpal region (El4) show average values of 29.2 °C and 30.0 °C. (**B**) Hypothermic newborn. In contrast to buffalo neonates with thermostability, this animal shows lower temperatures in both central and peripheral temperature. The temperature was lower in the periocular window (El1) by 1 °C and in the auricular region (El2) by 0.9 °C. In peripheral windows at the elbow (El3) and metacarpal region (El4), the temperature dropped by 0.7 °C and 2 °C, respectively. The significantly marked difference between the central and peripheral windows shows that an animal with hypothermia at birth generates microcirculatory changes to preserve body heat in the brain. This leads to the activation of the Autonomic Nervous System, its sympathetic branch, and the neurosecretion of catecholamines that cause peripheral vasoconstriction to prevent further heat loss. This would explain the response observed in the radiometric images of newborn buffaloes with hypothermia. Red triangles mark the maximum temperature and blue triangles the minimum values in each thermal window.

Studies by Santiago et al. [11] in 1260 Yorkshire-Landrace x large white piglets determined that a low surface temperature (assessed with an infrared thermographic camera) at birth is associated with low vitality scores. In this study, animals with low vitality scores were related to lower birth weight and lower temperatures (around 1 to 2 °C less than high-vitality piglets at birth and drying). At 24 h after birth, animals born from sows with parities 6 to 7 had higher vitality scores and temperatures (1.5 °C). In this sense, thermal imaging has been proposed as a non-invasive method to assess the thermal state of newborn animals [187]. Labeur et al. [187] studied the influence of shearing on the amount of brown fat tissue deposits in newborn lambs. Through infrared thermography in the dorsum of lambs subjected to cold challenge, the authors reported that shorn lambs had a higher core temperature after 30 min of a cold challenge than control animals. In a preliminary study conducted on piglets by the authors, infrared thermography showed that low surface temperatures at farrowing were associated with low birth weights, birth order, and mortality. In Figure 5, piglets from the same litter show different hindlimb temperatures. Moreover, Figure 6 shows the hypothermia effect in newborn water buffaloes.

In contrast, the evidence has led to questioning the benefit of using oxygen as a treatment for birth. Vande Pol et al. [188] evaluated the effect of drying and oxygen supply in 485 newborn piglets on rectal temperature during the first 24 h after birth. They observed that drying presented a greater benefit on temperature than using oxygen and drying together. Additionally, oxygen and drying did not affect the vitality or temperature of the piglets. This is possibly due to the obstruction by meconium aspiration, which intervenes in the process of oxygen exchange in the body [61]. An alternative to resolve this phenomenon has been suggested by allowing ventilatory assistance, as mentioned by Donnelly et al. [189], who have evaluated the effect of nasal oxygen insufflation and continuous positive airway pressure on pharmacologically induced respiratory suppression in 10 Holstein calves. They found that only insufflation with oxygen significantly increased pO_2_ and carbon dioxide (CO_2_), whereas oxygen administration with positive pressure reduced the presence of CO_2_, although there was no increase in pO_2_. This could indicate a possible improvement in the gas exchange phenomenon.

These results show that newborn mammals cannot completely thermoregulate during the first hours of life. Therefore, providing a suitable environment for the newborn at birth and during the first hours after parturition is essential to improve their vitality. For example, in piglets, although the farrowing houses are set at the recommended temperature for the sows (15–19 °C), this represents a challenge to the piglets that require an environmental temperature between 32 and 35 °C [190]. Moreover, although high temperatures are beneficial to newborn piglets, for sows, floor heating in periparturient sows with temperatures around 33 °C to 34 °C might cause a heat stress response [191]

A study analyzing the benefits of the creep area concerning mortality showed that enriching the area (e.g., insulated bedding and an additional wall to increase heat retention inside the creep) did not influence the mortality rate of animals (between 12.9 and 15.2% for all treatment groups) [192]. Milan et al. [190] evaluated the heat requirements for piglets when using heat lamps during the farrowing process by applying machine learning systems and the factors that can alter the quality of the supplemental heat. Increased air temperature (15–19 °C) and animal weight (between 1 and 2 kg) were two variables that altered the piglet heat requirement. Contrarily, heat lamps are only recommended for lambs while the animals are drying and should not be used after this [193].

Infrared thermography has helped to demonstrate that, apart from placing heat lamps, it is important to consider the lens, the shape, and the height of the lamps. For example, height affects the heated area and can increase/decrease hotspots, altering the net usable area for piglets to acquire their heat needs [194]. Automatic thermal control of heat lamps inside pig farms is being developed. These systems can be applied in newborns and piglets at different growth stages according to the already established temperatures to ensure thermal comfort. In another study, Pedersen et al. [195] applied seven thermal aids to evaluate their effects on reducing hypothermia in piglets on slatted or solid floors, and those with floor heating, a radiant heater from above, and provision of straw. When comparing the RT of the treatment piglets with control animals, all methods reduced the temperature loss and maintained piglets’ RT above 35 °C most of the time when providing straw and the radiant heater. In this way, using external heat sources depends on the species, individual elements, and the method characteristics to ensure a benefit for the offspring.

Another factor directly associated with the previous techniques is the thermal challenge due to the exposure to the extrauterine environment and the evaporative heat loss that neonates might present because they are born wet by the amniotic fluid and fetal membranes. After birth, farm animals like piglets are susceptible to chilling, hindering their vitality scores and other measures, such as low birth weight, environmental temperature, housing facilities, or delay in colostrum intake [70]. In the same species, decreases of 3.7 °C in RT during the first 30 min have been reported [188]. It is important to denote that sows do not lick the piglets to remove amniotic fluid; therefore, they remain wet for some hours and have more risk of hypothermia. Physiologically, maternal behaviors, such as licking the newborn immediately after birth, stimulate the offspring to stand, ingest colostrum, and dry their coat, reducing heat loss by evaporation [196]. Drying the newborn is a practice that is recommended in some cases.

Andersen et al. [197] studied the effect of drying newborn piglets or drying and placing animals under a heat lamp. Both treatments reduced the mortality (approximately 6% in both groups vs. 12% in control animals). Additionally, drying and heat lamp treatment resulted in fewer crushed piglets by their mothers (13.6% vs. 47.9% in control groups). This article shows that providing this type of perinatal care reduces the causes of mortality in newborns. Similarly, using a desiccant, a warming box (35 °C), and combining both methods and a control group have been studied [188]. The authors found that animals in the desiccant and the warming box had similar temperatures. However, combining both methods resulted in the highest RT between 10 and 120 min after farrowing (from 37.6 to 38.6 °C, respectively, vs. 36.7 to 37.7 °C in the control group), having the greatest effect on low-birth-weight piglets. Those findings mean that combining both methods reduces piglet temperature decline.

Other studies have shown that drying piglets with a desiccant increased RT up to 2.4 °C from 25 to 180 min after birth, reducing the decline in RT in piglets with low and high weights [198]. Likewise, in another study, Vasdal et al. [199] found that piglets dried and placed at the udder reduced postnatal mortality (by 10%). Therefore, combined techniques, such as colostrum supplementation, providing an external heat source, and a physical approach, are recommended to increase vitality in newborn mammals.

## 5. Perspectives

Study perspectives about the therapy to promote vitality in the newborn are focused on pharmacological therapies, such as caffeine and naloxone. Due to their direct cardiorespiratory effect, they could help to increase the availability of oxygen or blood resources [147]. However, the evidence does not indicate whether these effects may have a direct or positive relationship with increased vitality or decreased perinatal mortality. Likewise, it would be necessary to explore other drugs, such as sildenafil or doxapram, which have been suggested to have a similar mechanism of action to caffeine and naloxone, but with the advantage of having a cardiovascular effect, which would counteract hypertension, acidemia, and hypoxia during the postpartum period [200,201].

## 6. Conclusions

Proper assessment of vitality is essential for neonatal survival in farm animals. Assessing vitality immediately after birth is essential to determine the offspring’s health state and discover those requiring medical intervention to minimize the deleterious effect of intrapartum asphyxia.

Farms or veterinary clinics must train personnel and have adequate physical resources to assess the health and vitality of the newborn because a correct understanding of neonatal physiology is essential for interpreting vitality scores and detecting sick or weak newborns in need of corrective intervention. Vitality scores should reduce long-term neonatal morbidity and mortality in domestic animals. That is why Apgar scores in animals should be improved by expanding the use of neonatal reflexes, clinical examination, and blood gases. Biochemical and metabolic alterations explain the origin of cardiovascular and neurological abnormalities in newborns that survive intrapartum asphyxia. In addition, veterinarians must consider the type of delivery, the mother’s age, birth order, and birth weight when assessing newborn vitality.

It is also essential to use technologies applied to vitality assessment to provide important clinical data on fetal or newborn distress. It could be helpful in all farm and domestic species because it is not an invasive technique, and any person could apply it with basic training.

## Data Availability

Data sharing not applicable.
